# Supplementary material for: The relative resistance of children to sepsis mortality: from pathways to drug candidates
Source: Mol Syst Biol. 2018 May 17;14(5):e7998. doi: 10.15252/msb.20177998 (PMC5974511; doi:10.15252/msb.20177998)
Supplement: Supplementary file 1 — Appendix [file MSB-14-e7998-s001.pdf]

Appendix

Appendix Figure S1. DEGs from standard analysis of U133 arrays.

Appendix Table S1. Pathprint-PDN method: Drug curation references.

Appendix Table S2. DEGs-PDN method: Drug curation references.

Appendix Table S3. Random selection method: drug curation references.

Appendix Table S4. DEGs-LINCS method: Drug curation references.

Appendix Table S5. BarCode-LINCS method: Drug curation references.

Appendix References

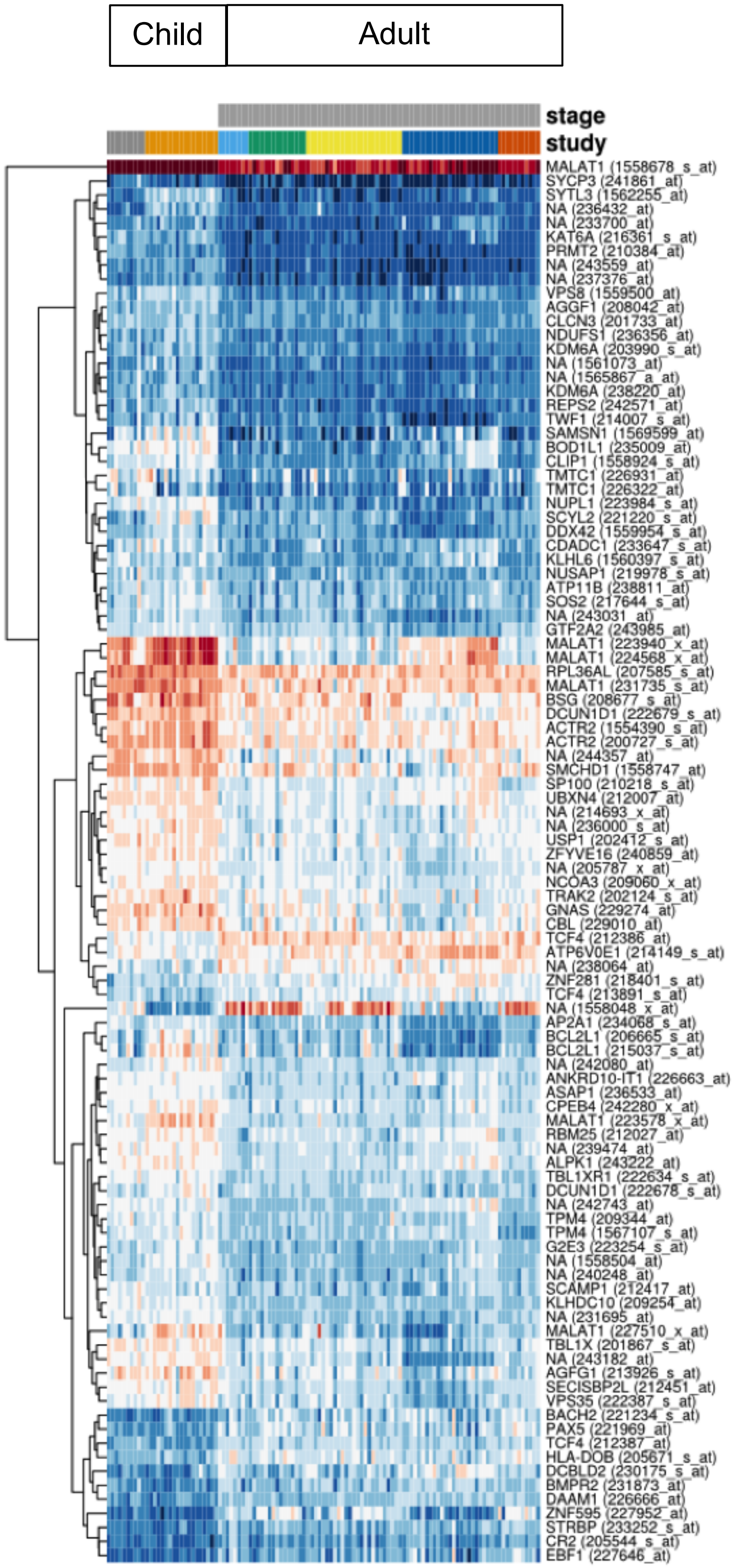

Appendix Figure S1 - DEGs from standard analysis of U133 arrays.
The figure above is a representative heat map of DEGs from standard limma analysis of all adult and child U133 Plus 2.0 microarrays. The heat-map colors red, white, and blue represent high, intermediate, and low expression respectively.

Pathprint-PDN Method	Benefit Reported	Harm Reported
fenoprofen	(Celik et al, 2002)	(Pettipher & Wimberly, 1994)
glibenclamide	(Zhang et al, 2014)	
asiaticoside	(Zhang et al, 2011)	
topiramate		
suramin	(Goto et al, 2006)	
hyoscyamine	(Fuentes et al, 2008)	
pancuronium		
N-acetyl-L-leucine		
mefenamicacid	(Celik et al, 2002)	(Pettipher & Wimberly, 1994)
apigenin	(Hu et al, 2016)	
camptothecin	(Rialdi et al, 2016)	
lincomycin	(Nameda et al, 2007)	
ganciclovir		
fursultiamine		
tocainide	(Fletcher & Ramwell, 1979)	
GW-8510		
tanespimycin	(Chatterjee et al, 2007)	
carbenoxolone	(Li et al, 2013)	
tacrolimus	(Jennings et al, 2009)	
conessine		
khellin		
eldeline		
sulfathiazole		
geldanamycin	(Chatterjee et al, 2007))	
cefoxitin		
procaine	(Fletcher & Ramwell, 1979)	
procyclidine		
monorden	(Chatterjee et al, 2007)	
hexetidine		
piperacetazine	(Villa et al, 1995)	
desipramine	(Brand et al, 2008)	
cyclosporine	(Larche et al, 2006)	
nifenazone	(Celik et al, 2002)	(Pettipher & Wimberly, 1994)
tanespimycin	(Chatterjee et al, 2007)	
etacrynicacid		
noscapine		
tanespimycin	(Chatterjee et al, 2007)	
mebhydrolin		
vincamine		
altretamine		
enalapril	(Gennari et al, 1996)	
coralyne	(Rialdi et al, 2016)	
napelline		
clindamycin	(Hirata et al, 2001)	

Appendix Table S1 - Pathprint-PDN method: Drug curation references.
A literature search using PubMed was performed to determine the number of therapeutic leads generated by the Pathprint-PDN drug prediction method that were shown to confer a survival benefit in *in vivo* mouse models of sepsis. The table above details the references for each drug with a reported benefit, reported harm, both, or none.

DEGs-PDN Method	Benefit Reported	Harm Reported
0297417-0002B		
indometacin	(Celik et al, 2002)	(Pettipher & Wimberly, 1994)
SB-202190	(Peng et al, 2003)	
acetohexamide	(Zhang et al, 2014)	
STOCK1N-35215		
emetine		
tacrine		
thioridazine		
suloctidil		
biotin		
cyclopenthiazide		
mebhydrolin	(Brackett et al, 1985)	
triprolidine	(Brackett et al, 1985)	
colchicine		
cinchonine		
methoxamine		
tanespimycin	(Chatterjee et al, 2007)	
fluorometholone	(Villa et al, 1995)	
nicardipine	(Bosson et al, 1986)	
quinpirole		
cicloheximide		
trimethylcolchicinic acid		
meteneprost		
puromycin		
digoxin		
naftidrofuryl		
terfenadine		
gelsemine		
sulindac	(Celik et al, 2002)	(Pettipher & Wimberly, 1994)
drofenine		
tioguanine		
methylergometrine		
methotrexate		
etacrynicacid		
dexamethasone	(Villa et al, 1995)	
tolazoline		
3-aminobenzamide	(Cuzzocrea et al, 1999)	
epitiostanol		
benzthiazide		
0179445-0000		
lidocaine	(Fletcher & Ramwell, 1979)	
alexidine		
dihydroergocristine		
nifurtimox		

Appendix Table S2 - DEGs-PDN method: Drug curation references.
A literature search using PubMed was performed to determine the number of therapeutic leads generated by the DEGs-PDN drug prediction method that were shown to confer a survival benefit in *in vivo* mouse models of sepsis. The table above details the references for each drug with a reported benefit, reported harm, both, or none.

Random Selection Method	Benefit Reported	Harm Reported
urapidil		
trifluoperazine		
metaraminol	(Spink & Vick, 1961)	
nomegestrol		
coralyne		
citiolone		
octopamine		
sulfapyridine		
butoconazole		
0175029-0000		
tracazolate		
tomatidine		
tetroquinone		
repaglinide		
tiletamine		
amikacin		
butirosin		
meptazinol	(Greeneltch et al, 2004)	
tolnaftate		
fasudil	(Ding et al, 2011)	
enilconazole		
sulfanilamide		
theophylline		
spiramycin		
omeprazole		
rolitetracycline		
dexpropranolol	(Wilson et al, 2013)	
piribedil		
sulfathiazole		
iobenguane		
dicycloverine		
PF-0053978-00		
dipivefrine		
aztreonam	(Machado et al, 2006)	
tomatidine		
bicuculline	(Hsu & Liu, 2004)	
ethosuximide		
meclozine		
alimemazine		
monensin		
Prestwick-691		
oxaprozin	(Celik et al, 2002)	(Pettipher & Wimberly, 1994)
amiodarone		
ampicillin		

Appendix Table S3 - Random selection method: drug curation references.
A literature search using PubMed was performed to determine the number of therapeutic leads generated by the Random selection drug prediction method that were shown to confer a survival benefit in *in vivo* mouse models of sepsis. The table above details the references for each drug with a reported benefit, reported harm, both, or none.

DEGs-LINCS Method	Benefit Reported	Harm Reported
IKK-inhibitor-X		
azacitidine	(Thangavel et al, 2014)	
primaquine		
cephaeline		
ZM-336372		
QL-X-138		
MBCQ		
XAV-939		
cycloheximide		
phenoxazine		
GSK-2334470		
tert-butylhydroquinone		
CGP-57380		
clofarabine		
verrucarin-a		
inhibitor-BEC		
CI-976		
fludarabine		
rucaparib		
emetine		
GSK-3-inhibitor-II		
resorcinol		
forskolin	(Yang et al, 2011)	
tyrphostin-AG-494		
cyproheptadine	(Moon, 1972)	
procaterol		
tunicamycin		
olaparib		
isoxsuprine		
calcipotriol		
BMS-641988		
tacrolimus	(Jennings et al, 2009)	
BML-259		
indirubin		
cladribine		
thiotepa		
navitoclax		
erastin		
ozagrel		
DC-45-A2		
PKCbeta-inhibitor		
RLM-2-12		
nifedipine	(Bosson et al, 1986)	
ruxolitinib	(Tsirigotis et al, 2015)	

Appendix Table S4 - DEGs-LINCS method: Drug curation references.
A literature search using PubMed was performed to determine the number of therapeutic leads generated by the DEGs-LINCS drug prediction method that were shown to confer a survival benefit in *in vivo* mouse models of sepsis. The table above details the references for each drug with a reported benefit, reported harm, both, or none.

BarCode-LINCS Method	Benefit Reported	Harm Reported
resveratrol	(Sebai et al, 2010)	
procaterol		
phenamil		
SD-6-035-A7		
fludarabine		
cyanoquinoline-11		
BIBU-1361		
NPC-15199		
dihydroergotamine		
simvastatin	(Merx et al, 2005)	
H-89		
ozagrel		
cyproheptadine		
calpeptin		
WZ-4-145		
galantamine		
promazine		
salubrinal		
AS-703026		
resorcinol		
cladribine		
salmeterol		
pyrrolidine-dithiocarbamate	(Nemeth et al, 1998)	
GR-127935		
ICI-199441		
ALW-II-38-3		
BIX-01294		
calyculin		
parthenolide		(Li et al, 2006)
thalidomide	(Noman et al, 2009)	(Ishikawa et al, 1998)
BRL-15572		
mibefradil		
prima-1-met		
elesclomol		
NSC-23766		
cytochalasin-b		
PKCbeta-inhibitor		
SJ-172550		
rimcazole		
GSK-3-inhibitor-II		
penicillic-acid		
temsirolimus	(Lee et al, 2010)	
metoclopramide		
RHO-kinase-inhibitor III[rockout]		

Appendix Table S5 - BarCode-LINCS method: Drug curation references.

A literature search using PubMed was performed to determine the number of therapeutic leads generated by the BarCode-LINCS drug prediction method that were shown to confer a survival benefit in *in vivo* mouse models of sepsis. The table above details the references for each drug with a reported benefit, reported harm, both, or none.

Appendix References

Bosson S, Kuenzig M, Schwartz SI (1986) Increased survival with calcium antagonists in antibiotic-treated bacteremia. *Circ Shock* **19:** 69-74

Brackett DJ, Schaefer CF, Wilson MF (1985) The effects of H1 and H2 histamine receptor antagonists on the development of endotoxemia in the conscious, unrestrained rat. *Circ Shock* **16:** 141-153

Brand V, Koka S, Lang C, Jendrossek V, Huber SM, Gulbins E, Lang F (2008) Influence of Amitriptyline on Eryptosis, Parasitemia and Survival of <i>Plasmodium Berghei</i>-Infected Mice. *Cell Physiol Biochem* **22:** 405-412

Celik I, Akbulut A, Kilic SS, Rahman A, Vural P, Canbaz M, Felek S (2002) Effects of ibuprofen on the physiology and outcome of rabbit endotoxic shock. *BMC Infect Dis* **2:** 26

Chatterjee A, Dimitropoulou C, Drakopanayiotakis F, Antonova G, Snead C, Cannon J, Venema RC, Catravas JD (2007) Heat shock protein 90 inhibitors prolong survival, attenuate inflammation, and reduce lung injury in murine sepsis. *Am J Respir Crit Care Med* **176:** 667-675

Cuzzocrea S, Zingarelli B, Costantino G, Sottile A, Teti D, Caputi AP (1999) Protective effect of poly(ADP-ribose) synthetase inhibition on multiple organ failure after zymosan-induced peritonitis in the rat. *Crit Care Med* **27:** 1517-1523

Ding RY, Zhao DM, Zhang ZD, Guo RX, Ma XC (2011) Pretreatment of Rho kinase inhibitor inhibits systemic inflammation and prevents endotoxin-induced acute lung injury in mice. *J Surg Res* **171:** e209-214

Fletcher JR, Ramwell PW (1979) Lidocaine treatment following baboon endotoxin shock improves survival. *Adv Shock Res* **2:** 219-232

Fuentes JM, Fulton WB, Nino D, Talamini MA, Maio AD (2008) Atropine treatment modifies LPS-induced inflammatory response and increases survival. *Inflamm Res* **57:** 111-117

Gennari R, Alexander JW, Boyce ST, Lilly N, Babcock GF, Cornaggia M (1996) Effects of the angiotensin converting enzyme inhibitor enalapril on bacterial translocation after thermal injury and bacterial challenge. *Shock* **6:** 95-100

Goto T, Takeuchi S, Miura K, Ohshima S, Mikami K-i, Yoneyama K, Sato M, Shibuya T, Watanabe D, Kataoka E, Segawa D, Endo A, Sato W, Yoshino R, Watanabe S (2006) Suramin prevents fulminant hepatic failure resulting in reduction of lethality through the suppression of NF-kappaB activity. *Cytokine* **33:** 28-35

Greeneltch KM, Haudenschild CC, Keegan AD, Shi Y (2004) The opioid antagonist naltrexone blocks acute endotoxic shock by inhibiting tumor necrosis factor-alpha production. *Brain Behav Immun* **18:** 476-484

Hirata N, Hiramatsu K, Kishi K, Yamasaki T, Ichimiya T, Nasu M (2001) Pretreatment of mice with clindamycin improves survival of endotoxic shock by modulating the release of inflammatory cytokines. *Antimicrob Agents Chemother* **45:** 2638-2642

Hsu DZ, Liu MY (2004) Bicuculline methiodide attenuates hepatic injury and decreases mortality in septic rats: role of cytokines. *Shock* **22:** 347-350

Hu W, Wang X, Wu L, Shen T, Ji L, Zhao X, Si CL, Jiang Y, Wang G (2016) Apigenin-7-O-beta-D-glucuronide inhibits LPS-induced inflammation through the inactivation of AP-1 and MAPK signaling pathways in RAW 264.7 macrophages and protects mice against endotoxin shock. *Food Funct* **7:** 1002-1013

Ishikawa M, Kanno S, Takayanagi M, Takayanagi Y, Sasaki K (1998) Thalidomide promotes the release of tumor necrosis factor-alpha (TNF-alpha) and lethality by lipopolysaccharide in mice. *Biol Pharm Bull* **21:** 638-640

Jennings C, Kusler B, Jones PP (2009) Calcineurin inactivation leads to decreased responsiveness to LPS in macrophages and dendritic cells and protects against LPS-induced toxicity in vivo. *Innate Immun* **15:** 109-120

Larche J, Lancel S, Hassoun SM, Favory R, Decoster B, Marchetti P, Chopin C, Neviere R (2006) Inhibition of mitochondrial permeability transition prevents sepsis-induced myocardial dysfunction and mortality. *J Am Coll Cardiol* **48:** 377-385

Lee PS, Wilhelmson AS, Hubner AP, Reynolds SB, Gallacchi DA, Chiou TT, Kwiatkowski DJ (2010) mTORC1-S6K activation by endotoxin contributes to cytokine up-regulation and early lethality in animals. *PLoS One* **5:** e14399

Li W, Li J, Sama AE, Wang H (2013) Carbenoxolone blocks endotoxin-induced protein kinase R (PKR) activation and high mobility group box 1 (HMGB1) release. *Mol Med* **19:** 203-211

Li X, Cui X, Li Y, Fitz Y, Hsu L, Eichacker PQ (2006) Parthenolide has limited effects on nuclear factor-kappa beta increases and worsens survival in lipopolysaccharide-challenged C57BL/6J mice. *Cytokine* **33:** 299-308

Machado DP, Nunes FB, Simoes Pires MG, D'Avila LC, Leite CE, Ruschel RE, da Cunha AA, Saciura VC, Poloni JA, Lunardelli A, de Oliveira JR, Alves Filho JC, Cunha FQ, Dias FS, Poli de Figueiredo CE (2006) Effects of beta-lactam antibiotics and L-arginine in the treatment of experimental sepsis in rats. *Int J Antimicrob Agents* **28:** 478-480

Merx MW, Liehn EA, Graf J, van de Sandt A, Schaltenbrand M, Schrader J, Hanrath P, Weber C (2005) Statin treatment after onset of sepsis in a murine model improves survival. *Circulation* **112:** 117-124

Moon RJ (1972) Carbohydrate metabolism and survival of endotoxin-poisoned mice given tryptophan. *Infect Immun* **5:** 288-294

Nameda S, Miura NN, Adachi Y, Ohno N (2007) Lincomycin protects mice from septic shock in beta-glucan-indomethacin model. *Biol Pharm Bull* **30:** 2312-2316

Nemeth ZH, Hasko G, Vizi ES (1998) Pyrrolidine dithiocarbamate augments IL-10, inhibits TNF-alpha, MIP-1alpha, IL-12, and nitric oxide production and protects from the lethal effect of endotoxin. *Shock* **10:** 49-53

Noman AS, Koide N, Khuda, II, Dagvadorj J, Tumurkhuu G, Naiki Y, Komatsu T, Yoshida T, Yokochi T (2009) Thalidomide inhibits lipopolysaccharide-induced nitric oxide production and prevents lipopolysaccharide-mediated lethality in mice. *FEMS Immunol Med Microbiol* **56:** 204-211

Peng T, Lu X, Lei M, Moe GW, Feng Q (2003) Inhibition of p38 MAPK decreases myocardial TNF-alpha expression and improves myocardial function and survival in endotoxemia. *Cardiovasc Res* **59:** 893-900

Pettipher ER, Wimberly DJ (1994) Cyclooxygenase inhibitors enhance tumour necrosis factor production and mortality in murine endotoxic shock. *Cytokine* **6:** 500-503

Rialdi A, Campisi L, Zhao N, Lagda AC, Pietzsch C, Ho JSY, Martinez-Gil L, Fenouil R, Chen X, Edwards M, Metreveli G, Jordan S, Peralta Z, Munoz-Fontela C, Bouvier N, Merad M, Jin J, Weirauch M, Heinz S, Benner C et al (2016) Topoisomerase 1 inhibition suppresses inflammatory genes and protects from death by inflammation. *Science* **352:** aad7993

Sebai H, Sani M, Ghanem-Boughanmi N, Aouani E (2010) Prevention of lipopolysaccharide-induced mouse lethality by resveratrol. *Food Chem Toxicol* **48:** 1543-1549

Spink WW, Vick JA (1961) Reversal of experimental endotoxin shock with a combination of aldosterone and metaraminol. *Proc Soc Exp Biol Med* **107:** 777-779

Thangavel J, Malik AB, Elias HK, Rajasingh S, Simpson AD, Sundivakkam PK, Vogel SM, Xuan Y-T, Dawn B, Rajasingh J (2014) Combinatorial therapy with acetylation and methylation modifiers attenuates lung vascular hyperpermeability in endotoxemia-induced mouse inflammatory lung injury. *Am J Pathol* **184:** 2237-2249

Tsirigotis P, Papanikolaou N, Elefanti A, Konstantinou P, Gkirkas K, Rontogianni D, Siafakas N, Karakitsos P, Roilides E, Dimitriadis G, Zerva L, Meletiadis J (2015) Treatment of Experimental Candida Sepsis with a Janus Kinase Inhibitor Controls Inflammation and Prolongs Survival. *Antimicrob Agents Chemother* **59:** 7367-7373

Villa P, Sartor G, Angelini M, Sironi M, Conni M, Gnocchi P, Isetta AM, Grau G, Buurman W, van Tits LJ (1995) Pattern of cytokines and pharmacomodulation in sepsis induced by cecal

ligation and puncture compared with that induced by endotoxin. *Clin Diagn Lab Immunol* **2:** 549-553

Wilson J, Higgins D, Hutting H, Serkova N, Baird C, Khailova L, Queensland K, Vu Tran Z, Weitzel L, Wischmeyer PE (2013) Early propranolol treatment induces lung heme-oxygenase-1, attenuates metabolic dysfunction, and improves survival following experimental sepsis. *Crit Care* **17:** R195

Yang W, Qiang D, Zhang M, Ma L, Zhang Y, Qing C, Xu Y, Zhen C, Liu J, Chen YH (2011) Isoforskolin pretreatment attenuates lipopolysaccharide-induced acute lung injury in animal models. *Int Immunopharmacol* **11:** 683-692

Zhang LN, Zheng JJ, Zhang L, Gong X, Huang H, Wang CD, Wang B, Wu MJ, Li XH, Sun WJ, Liu YJ, Wan JY (2011) Protective effects of asiaticoside on septic lung injury in mice. *Exp Toxicol Pathol* **63:** 519-525

Zhang W, Xu X, Kao R, Mele T, Kvietys P, Martin CM, Rui T (2014) Cardiac Fibroblasts Contribute to Myocardial Dysfunction in Mice with Sepsis: The Role of NLRP3 Inflammasome Activation. *PLoS ONE* **9:** e107639
