# Supplementary material for: The relative resistance of children to sepsis mortality: from pathways to drug candidates
Source: Mol Syst Biol. 2018 May 17;14(5):e7998. doi: 10.15252/msb.20177998 (PMC5974511; doi:10.15252/msb.20177998)
Supplement: Supplementary file 2 — Expanded View Figures PDF [file MSB-14-e7998-s002.pdf]

Expanded View Figures

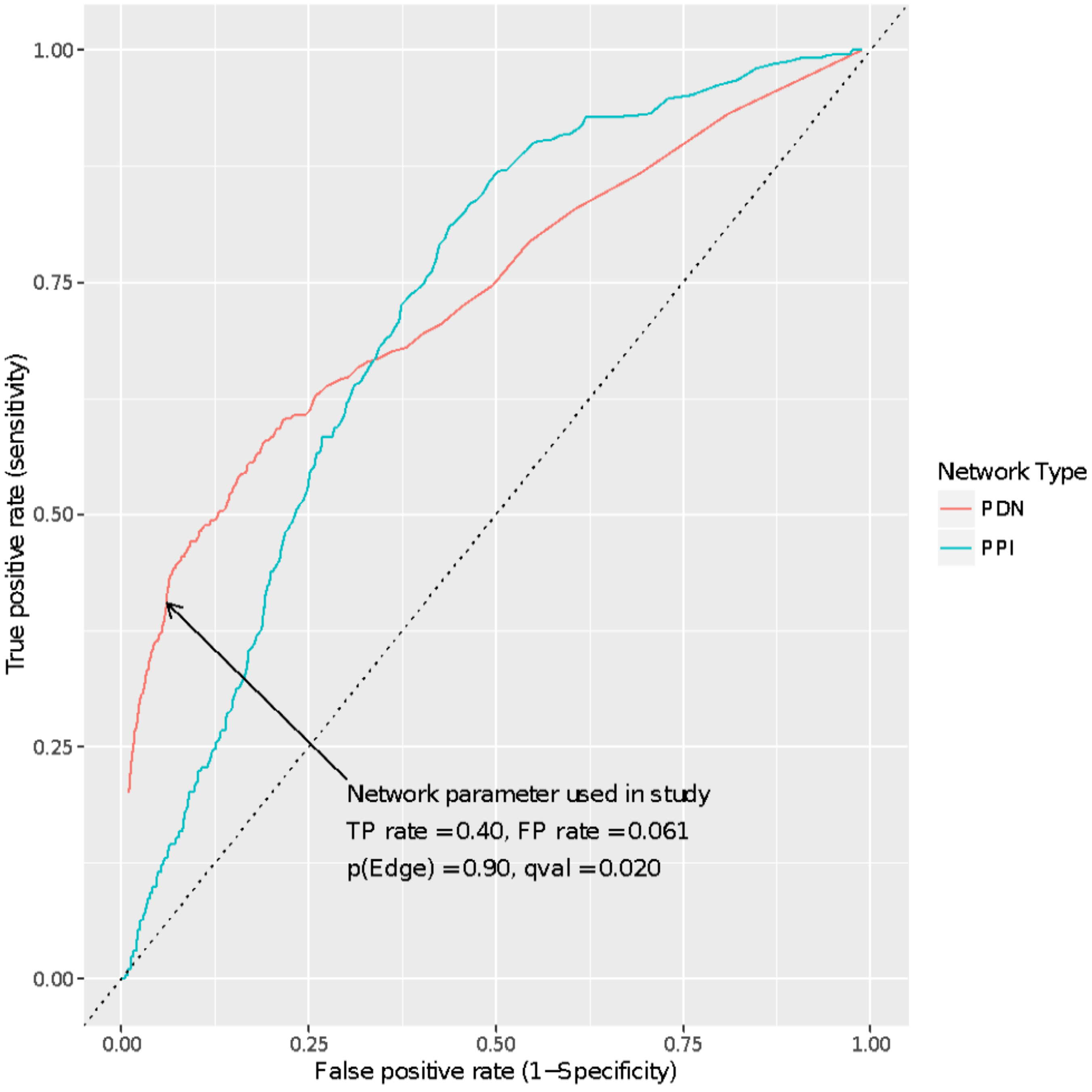

Figure EV1. Benchmarking: PDN and PPI Sensitivity vs. Specificity.

To provide a benchmark for new PDN methodology, we compared drug–disease relationships produced using PDNs with curated, known drug–disease relationships from the NDFRT and SPL databases. The true-positive (TP) and false-positive (FP) rates (Sensitivity and 1-Specificity) of the PDN predictions were compared to those generated using an alternative approach based on gene-level curated protein–protein interactions (PPI). The arrow points to the network cutoff parameters used in the study: TP rate, FP rate, pEdge (probability that there is an edge between any pair of nodes), and qval (*q*-value or FDR).

 © 2018 The Authors

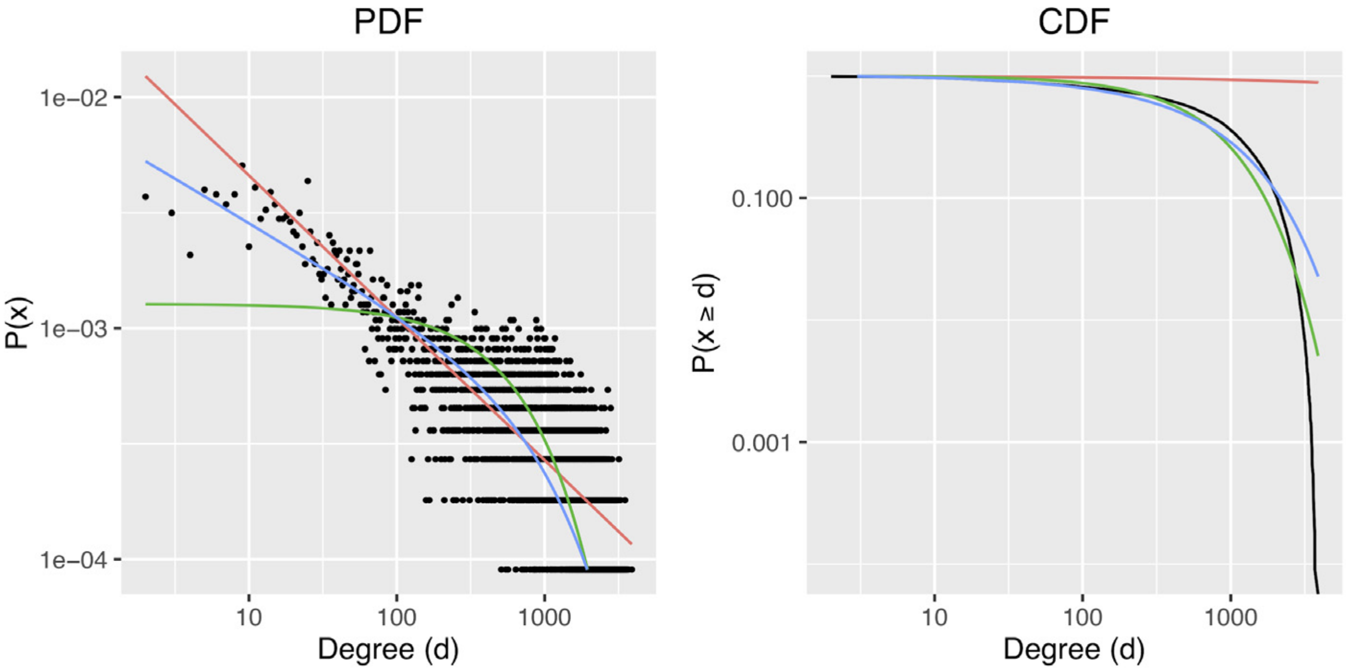

Figure EV2. PDN degree distribution.

The degree distribution of the PDN plotted as a probability density function (PDF) and cumulative density function (CDF). The data are shown in black, together with fits to power law (red), exponential (green), and power law with exponential cutoff (blue) distributions.

© 2018 The Authors
